# The Impact of the Orthodontic Forces on the Internal Resorptive Process for Intact Periodontium: A Finite Element Analysis

**DOI:** 10.3390/jcm15093335

**Published:** 2026-04-27

**Authors:** Radu-Andrei Moga, Cristian Doru Olteanu, Ada Gabriela Delean

**Affiliations:** 1 Department of Odontology, Endodontics and Oral Pathology, School of Dental Medicine, University of Medicine and Pharmacy Iuliu Hatieganu, Str. Motilor 33, 400001 Cluj-Napoca, Romania; ada.delean@umfcluj.ro; 2 Department of Orthodontics, School of Dental Medicine, University of Medicine and Pharmacy Iuliu Hatieganu, Str. Avram Iancu 31, 400083 Cluj-Napoca, Romania

**Keywords:** orthodontically induced internal root resorption, intact periodontium, orthodontic movements, finite elements analysis, failure criteria

## Abstract

**Background/Objectives**: This numerical (finite element analysis/FEA) study aimed to analyze the internal stress distribution patterns caused by a 4 N orthodontic force during intrusion, extrusion, rotation, tipping, and translation, using four common failure criteria, in intact periodontium. Additionally, based on these stress patterns, the study sought to establish correlations between these failure criteria to determine the most appropriate one—brittle-like or ductile-like. The orthodontically induced internal resorption was also assessed, along with the influence of orthodontic movements on the topography of the resorptive processes. **Methods**: A total of 180 numerical simulations on nine 3D anatomically accurate models containing the second lower premolar (manually reconstructed, CBCT-based) were performed. The brittle-like Maximum Principal, Minimum Principal, and ductile-like Von Mises and Tresca criteria were employed for the numerical analyses. **Results**: Translation and rotation more frequently cause internal pulp chamber resorption (vestibular, occlusal, lingual–mesial walls). In rotation, the stress was directly caused by the force applied to the bracket, while in translation, the origin of the stress was from the lingual third cervical area. Intrusion and extrusion movements are most likely to cause resorption in the root canal’s cervical and middle thirds (vestibular and proximal walls) due to high stresses induced by movement at the external cervical vestibular region. Tipping seems to be least prone to internal resorption. **Conclusions**: A 4 N orthodontic force can induce internal resorption in the pulp chamber and in the middle and cervical thirds of the root canals. The ductile-like failure criteria appear to provide a more accurate assessment of internal orthodontically induced resorption than the brittle-like criteria.

## 1. Introduction

Orthodontic treatment (i.e., movements [1,2]) can induce unpredictable islands of external and internal orthodontic resorption, induced by the circulatory disturbances, applied force variations in the maximum local hydrostatic pressure, and stress distribution in the dental tissues [3]. The prevalence of these processes is reported to be variable, starting from 0.02 to 14.8% [4,5,6,7] external cervical and radicular and 1–51.5% [5] internal radicular, rising up to 20–100% external cervical, along with other reports [8,9,10]. The resorptive processes involve the dentine and cementum of both the root and the crown [5,8,11]. Nevertheless, a proper diagnosis of the origin of the resorptions (e.g., orthodontic vs. occlusal trauma) should be made, since it will highly influence the tissular biomechanical behavior, as reported in previous numerical studies assessing the external [4,12,13,14,15,16,17,18,19] and internal [20] responsive processes.

The orthodontic resorption pathological mechanism is not completely understood (especially that of the internal process), and if not rapidly identified and stopped, it will become irreversible, with consequences for the outcome of the orthodontic treatment, and it might involve additional dental treatment [21,22,23,24,25,26,27,28,29,30,31,32] for repair [4,5,7,33]. Nevertheless, since it is usually asymptomatic, it is extremely difficult to identify, and by the time the islands of resorption are clinically visible, the process has already become irreversible [5,8,11,34,35,36,37,38,39,40]. Finite element analysis (FEA) represents a valuable tool for investigating the stress distribution within dental tissues, since it allows us to see the individual stress distribution in each tissular component, as well as the origin of the induced stress [2,12,13,34,35,36,37,38,41,42,43,44]. Since the applied orthodontic force will induce stress that will be absorbed and dissipated through the entire tooth structure [34,35,36,37,38], there are reports regarding the optimal amount of force considered to be relatively safe, 0.28–3.31 N [45,46,47]; however, there is no consensus regarding this issue [34,35,36,37,38,48]. In a recent report regarding the external orthodontic root resorption, 4 N were analyzed, with only a few localized islands being prone to resorption [48]. Nevertheless, there are only a handful of numerical analyses investigating the orthodontically induced external resorption, and even fewer investigating internal resorption [48]. Most of the numerical analyses were focused on simulated occlusal trauma-induced resorptive processes (e.g., abfraction [4,12,13,14,49]), which significantly modify the stress absorption distribution in the dental tissues [10,15,16,19,34,50,51,52,53]. These analyses reported cervical third resorption to be induced by extrusion, middle third lacunae to be induced by rotation, and apical third resorption to be induced by intrusion and extrusion [6,10,21,22,23,24,25,26,27,28,29,30,31,32,52,53,54,55].

Moreover, these numerical analyses reported numerous inconsistencies, both qualitative (stress display) and quantitative (numerical results), contradicting the clinical reports as well as the known normal biomechanical behavior of the dental tissues [3,10,45,46,47,50,51,52,53]. These inconsistencies [50,51,52,53] are related to the fact that the numerical method, being a widely used method in the engineering field [56], requires certain conditions for providing accurate results. Thus, besides the anatomically accurate 3D models and boundary assumptions, the failure criteria describing the biomechanical behavior of the analyzed tissues must be consistent with the type of analyzed material. No numerical studies followed the above-mentioned; thus, there are accuracy issues [50,51,52,53]. It must be acknowledged that dental tissues behave like ductile-like materials [42,57,58,59,60]; thus, only Von Mises and Tresca failure criteria can provide accurate results [34,35,36,37,38]. Nevertheless, most of the dental numerical studies employed brittle-like criteria or even the hydrostatic pressure ones [45,46,47,50,51,52,53], which will lead to a less accurate behavioral description [34,35,36,37,38]. There are even numerical studies that clearly stated that dentine is a brittle-like structure [61], despite the clinical data and anatomical micro-architecture [34,42,59,60,61,62], favoring the Maximum Principal/MaxP and Minimum Principal/MinP [61].

This study aimed to analyze the internal stress distribution patterns caused by a 4 N orthodontic force during intrusion, extrusion, rotation, tipping, and translation in lower premolars with intact periodontium, using the four most common failure criteria. Additionally, based on these stress patterns, the study sought to establish correlations between these failure criteria to determine the most appropriate one—brittle-like or ductile-like. The orthodontic-induced internal resorption was also assessed, along with the influence of orthodontic movements on the topography of the resorptive processes.

## 2. Materials and Methods

Our manuscript is the continuation of an ongoing stepwise numerical study (nr. 158/2 April 2018) assessing dental tissular biomechanical behavior under orthodontic movements, various loads, and bone-loss levels. The study included nine patients (mean age of 29.81 ± 1.45 years, 4 males and 5 females) with an indication for orthodontic treatment. Each patient had a CBCT (cone-beam computed tomography, ProMax 3DS, Planmeca, Helsinki, Finland, voxel size 0.075 mm) scan for therapeutic reasons. The area of the second lower premolar and surrounding teeth was selected. The inclusion criteria for the study were related to non-inflamed periodontium, intact periodontium, or bone loss up to 2 mm, no mandibular tooth loss, intact teeth, no malposition, an indication for orthodontic treatment, and willingness for regular monitoring.

**Figure 1:**
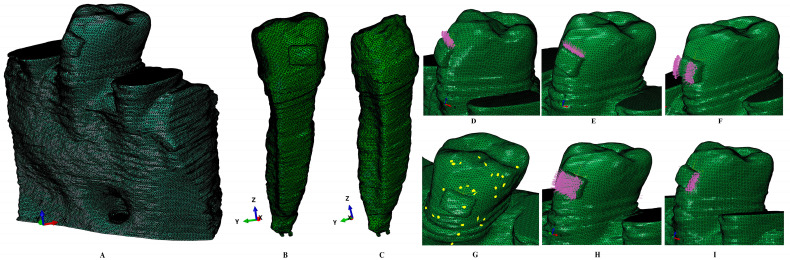
Mesh model of one of the nine 3D models: (**A**) 2nd lower right premolar model with intact periodontium, (**B**) tooth structure with enamel, dentine, dental pulp, NVB, and stainless-steel bracket, (**C**) dentine structure with dentine, dental pulp, and NVB, (**D**) applied vectors—extrusion, (**E**) applied vectors—intrusion, (**F**) applied vectors—rotation, (**G**) enamel, dentine, pulp chamber, pulp and stainless-steel bracket mesh with warning elements, (**H**) applied vectors—tipping, (**I**) applied vectors—translation.

Based on the radiological images, 3D models of the dental tissues were manually reconstructed using Amira 5.4.0. (Visage Imaging Inc., Andover, MA, USA), obtaining nine 3D models that were anatomically accurate, containing the second lower premolar. This choice was made based on the scarce numerical studies related to this tooth, as well as its topographical importance for force distribution during orthodontic treatment. In each model, the enamel, dentine, dental pulp, apical neuro-vascular bundle/NVB, periodontal ligament/PDL, cortical, and trabecular bone were selected, reconstructed, and assembled into the 3D models (Figure 1). The missing bone was manually reconstructed as close as possible to the anatomical reality. The cementum could not possibly be differentiated from dentine, so due to similar physical properties (Table 1), it was reconstructed as dentine. The PDL has a variable thickness of 0.15–0.22 mm. The alveolar socket of the neighboring teeth was filled with cortical and trabecular bones. The base of a stainless-steel bracket was reconstructed on the enamel structure.

The global element size of the mesh was 0.08–0.116 mm and the number of C3D4 tetrahedral elements was 5.06–6.05 million, while the number of nodes was 0.97–1.07 million. There were no element errors, and only surface element warnings (e.g., Figure 1, for 6.05 million elements, 264 element warnings were signaled, approx. 0.0043% of the total number of elements). Mesh convergence tests were performed.

The numerical analysis (FEA) was performed using ABAQUS 6.13-1 software (Dassault Systèmes Simulia Corp., Maastricht, The Netherlands), by employing four of the most commonly used failure criteria in the dental studies. Thus, the brittle-like Maximum Principal/MaxP tensile and Minimum Principal/MinP compressive stresses, and the ductile-like Von Mises/VM overall and Tresca/T shear stresses were used. The applied force was 4 N, being selected to assess the biomechanical behavior of dental tissues under medium–large orthodontic forces used for the clinical phase. The movements selected were intrusion, extrusion, rotation, tipping, and translation. The study totaled 180 numerical simulations. The analyzed structures were the entire tooth containing bracket base–enamel–dentine–dental pulp–NVB, and a dentine structure containing dentine and dental pulp. As in the other dental numerical studies, the boundary assumptions were isotropy, linear elasticity, homogeneity, perfectly bonded interfaces, and zero displacements for the base of the models.

The results included quantitative numerical values and a qualitative color-coded stress display. The various color shades were seen as high (red-orange), medium (yellow-green), and low (blue). Correlations with other dental numerical analyses were performed.

## 3. Results

**Figure 2:**
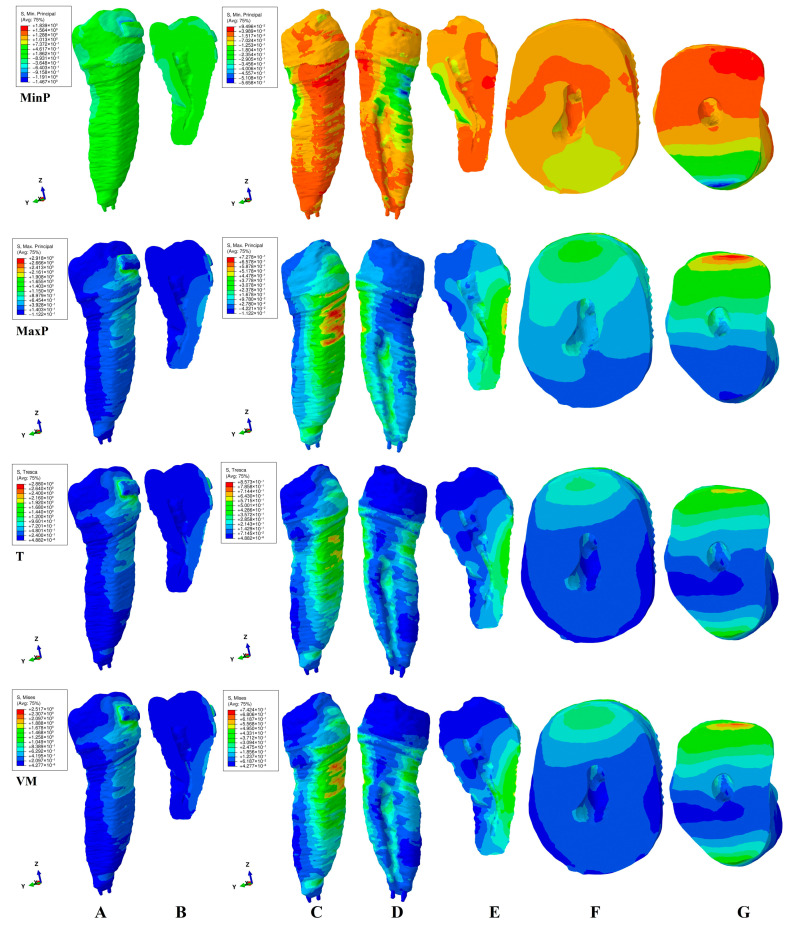
Internal and external stress display (in MPa/1 KPa = 0.001 MPa = 1.00 × 10^−3^ MPa) during extrusion in tooth ((**A**) distal–vestibular view and (**B**) proximal vertical section) and dentine ((**C**) distal–vestibular, (**D**) mesial–lingual, (**E**) proximal vertical section, (**F**) horizontal crown section at bracket level, (**G**) horizontal root section in cervical third) structures using the four failure criteria: MinP—Minimum Principal (compressive), MaxP—Maximum Principal (tensile), T—Tresca (shear), and VM—Von Mises (overall).

**Figure 3:**
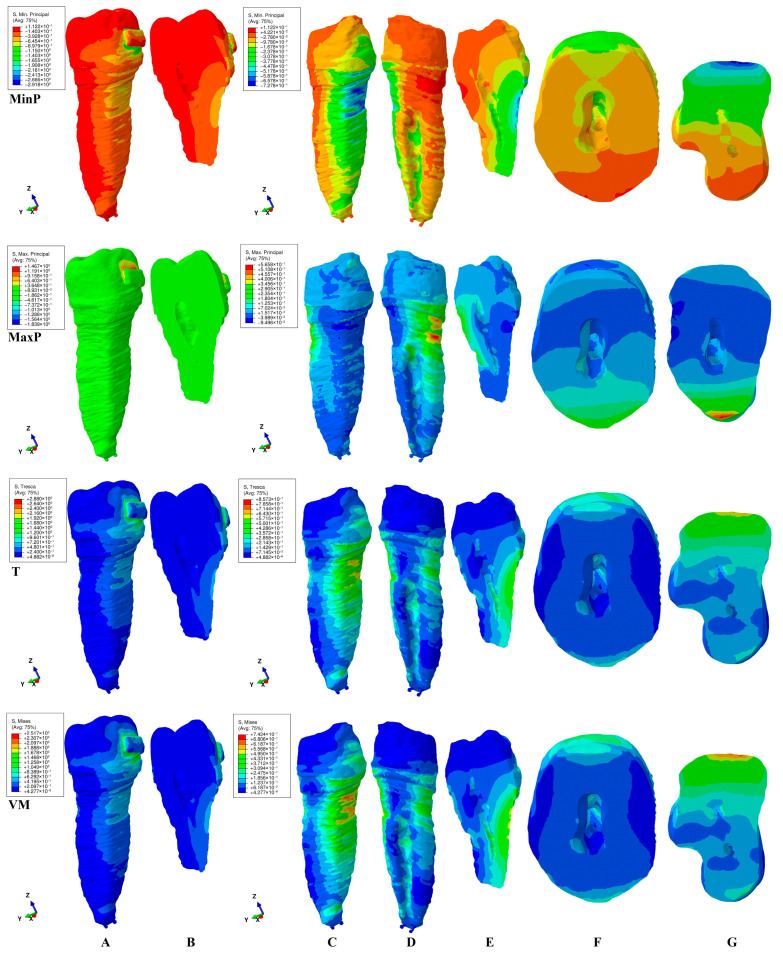
Internal and external stress display (in MPa/1 KPa = 0.001 MPa = 1.00 × 10^−3^ MPa) during intrusion in tooth ((**A**) distal–vestibular view and (**B**) proximal vertical section) and dentine ((**C**) distal–vestibular, (**D**) mesial–lingual, (**E**) proximal vertical section, (**F**) horizontal crown section at bracket level, (**G**) horizontal root section in cervical third) structures using the four failure criteria: MinP—Minimum Principal (compressive), MaxP—Maximum Principal (tensile), T—Tresca (shear), and VM—Von Mises (overall).

**Figure 4:**
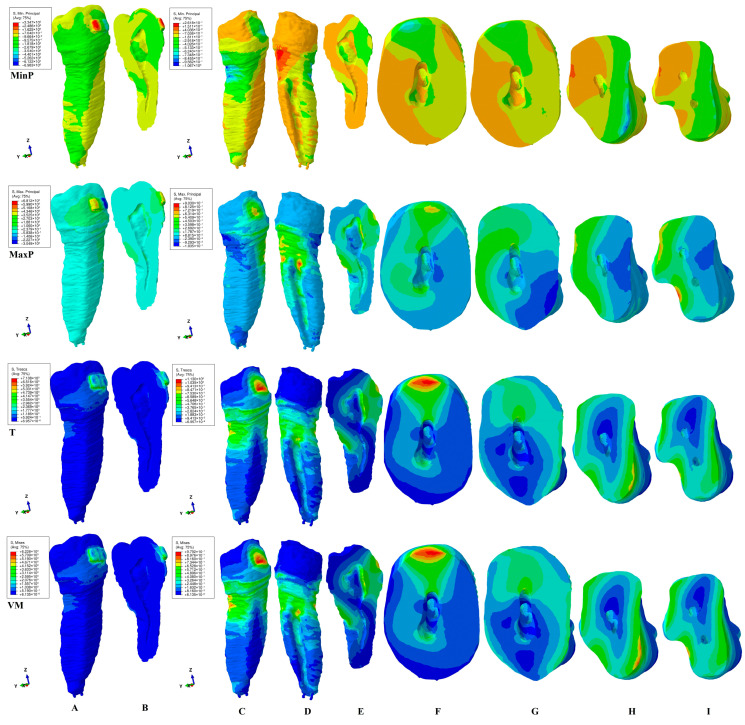
Internal and external stress display (in MPa/1 KPa = 0.001 MPa = 1.00 × 10^−3^ MPa) during rotation in tooth ((**A**) distal–vestibular view and (**B**) proximal vertical section) and dentine ((**C**) distal–vestibular, (**D**) mesial–lingual, (**E**) proximal vertical section, (**F**,**G**) horizontal crown section at bracket level, (**H**,**I**) horizontal root section in cervical third) structures using the four failure criteria: MinP—Minimum Principal (compressive), MaxP—Maximum Principal (tensile), T—Tresca (shear), and VM—Von Mises (overall).

**Figure 5:**
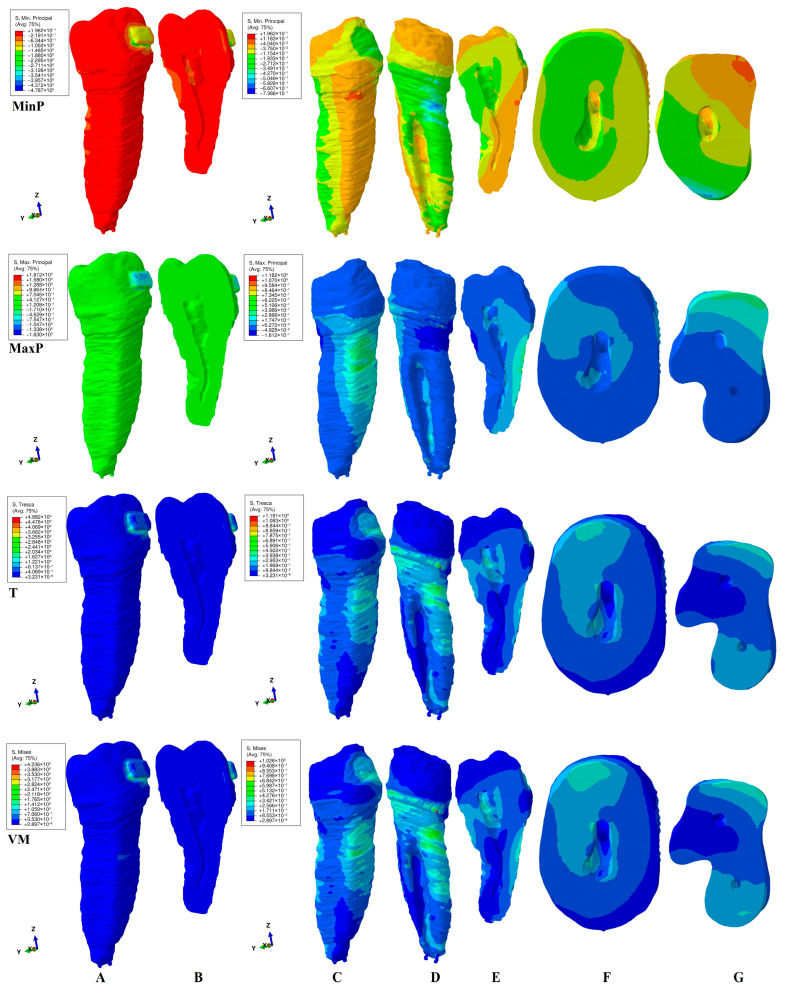
Internal and external stress display (in MPa/1 KPa = 0.001 MPa = 1.00 × 10^−3^ MPa) during tipping in tooth ((**A**) distal–vestibular view and (**B**) proximal vertical section) and dentine ((**C**) distal–vestibular, (**D**) mesial–lingual, (**E**) proximal vertical section, (**F**) horizontal crown section at bracket level, (**G**) horizontal root section in cervical third) structures using the four failure criteria: MinP—Minimum Principal (compressive), MaxP—Maximum Principal (tensile), T—Tresca (shear), and VM—Von Mises (overall).

**Figure 6:**
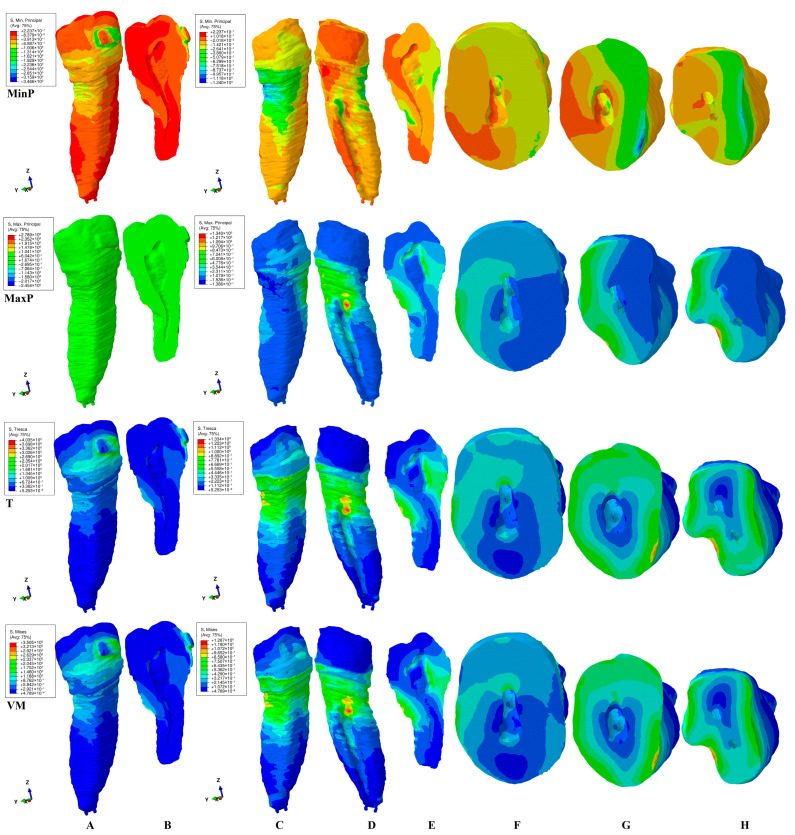
Internal and external stress display (in MPa/1 KPa = 0.001 MPa = 1.00 × 10^−3^ MPa) during translation in tooth ((**A**) distal–vestibular view and (**B**) proximal vertical section) and dentine ((**C**) distal–vestibular, (**D**) mesial–lingual, (**E**) proximal vertical section, (**F**) horizontal crown section at bracket level, (**G**,**H**) horizontal root section in cervical third) structures using the four failure criteria: MinP—Minimum Principal (compressive), MaxP—Maximum Principal (tensile), T—Tresca (shear), and VM—Von Mises (overall).

This study investigated the internal stress display in the dental pulp chamber and root canals of a second lower premolar in 180 FEA simulations using four failure criteria. The internal stress display was correlated with the external stress display in both the tooth (bracket–enamel–dentine–pulp–NVB) and dentine (dentine–pulp–NVB) structures to better assess the biomechanical behavioral stress distribution. The qualitative (Figure 2, Figure 3, Figure 4, Figure 5 and Figure 6, MPa) and quantitative (Table 2, KPa) results were presented, and their biomechanical accuracy was evaluated through correlation.

The quantitative results (Table 2, KPa) showed that the highest amounts of internal stress were displayed by the translation, closely followed by rotation in the coronal pulpal chamber, which was almost double that of the external coronal stresses. The internal stress in both pulp and root canals was lower than the external stress. The single exception was the translation, where the internal pulp chamber wall stress (due to the internal absorption–dissipation pattern) was almost double that of the external coronal one, but lower than the external cervical one (which induced it). Among the five movements, tipping displayed the lowest amount of internal stress. For translation and rotation, the highest amount of stress was seen in the cervical third of the root canal and pulp chamber. For intrusion and extrusion, the highest amounts were displayed in the cervical third of the root canal. The numerical inconsistencies were seen for tipping (MaxP, internal pulp chamber stress higher than external one) and for extrusion (MinP, internal pulp chamber stress higher and with a negative sign, vs. lower and positive sign external stress). VM and T showed no sign of such inconsistencies. Nevertheless, a clear differentiation between brittle and ductile-like was not possible.

Figure 2, Figure 3, Figure 4, Figure 5 and Figure 6 displayed both external stress (tooth and dentine, A, B, and C), as well as the internal dentine stress (tooth, B, and dentine D–I). The internal stress was visible only when the dentine structure was analyzed for all four criteria. Some inconsistencies related to the external stress display were visible for the brittle-like MinP (extrusion, tipping) and MaxP (intrusion, rotation, tipping, translation), the color-coded stress display. Based on the color-coded stress display, differentiations among brittle and ductile-like criteria were possible.

*Extrusion* (Figure 2). Qualitatively, in the dentine structure, all four criteria (i.e., diverse types of stress) displayed the same color-coded coronal pulp chamber area (lingual–mesial and occlusal walls), showing potential islands of internal resorption (Figure 2E–G). It appears that the external stress in the cervical third of the root spread to the areas mentioned above. Additionally, VM, T, and MaxP showed internal root canal stress in the cervical and middle thirds, mainly on the vestibular wall/side. However, a closer examination revealed some differences. Tresca and Von Mises failure criteria exhibited similar stress patterns on both the internal and external dentine surfaces. The Von Mises criteria displayed more extensive red-orange areas of external cervical stress than Tresca, likely due to design differences. Internal stress within the pulp chamber on the proximal side was caused by external stress on the cervical third (Figure 2C,E,F). The stress in the root canal’s cervical and middle thirds was highest (green), generated by and dissipating through the external dentinal surface. The Maximum principal tensile stress revealed a distinct pattern, with lower stress in the coronal region and higher stress in the cervical and middle thirds of the root canal. The spread of the tensile stress was more intense than that of the overall and shear stress, as shown in Figure 2F,G. VM, T, and MaxP displayed similar stress patterns on the vestibular side of both the tooth and dentine structures. The Minimum principal compressive stress was highest on the lingual sides, with internal manifestations on the same side, but less intense than the other three stresses. Nonetheless, significant differences existed between stress in the tooth structure (indicating more tension) and the external dentine surface (more accurately showing compression areas) for the MinP. The higher external and internal stresses that were prone to inducing external and internal orthodontic resorption were limited in depth (Figure 2F–G), which was consistent with known clinical biomechanical behavior. The MaxP internal and external stress spreads, however, were too extensive from a biomechanical point of view, while the MinP displayed the color-coded inconsistencies (tooth vs. dentine structures), with both signaling lower accuracy. Quantitatively, MinP showed coronal stress inconsistencies, with the internal stress being higher than the external, which was biomechanically incorrect.

*Intrusion* (Figure 3). Qualitatively, the external stress distribution in both the tooth and dentine structure displayed similar color-coded stresses under Tresca and Von Mises criteria as extrusion movement (Figure 2A,C,D vs. Figure 3A,C,D). However, when analyzing the internal stress display, noticeable differences were seen (Figure 2E,F,G vs. Figure 3E,F,G). The pulp chamber internal stress involved the same lingual–mesial and occlusal walls, but with a smaller extent than extrusion. The root canal internal stress guarded the same vestibular-proximal middle and cervical thirds display and color (green). The depth of VM and T’s highest cervical third stress (red-orange) was similar to extrusion, and it was small, Figure 3F,G (similar to the clinical biomechanical behavior). Noticeable differences were seen for the MinP and MaxP criteria, due to the particular type of stress investigation. The MaxP displayed almost no tensile stress in the tooth structure, but there was visible concentrated high red-orange lingual cervical stress in the dentine structure. These inconsistencies were related to the assessed type of stress. The pulp chamber displayed the same lingual–mesial and occlusal stress as in extrusion, but it was induced by the lingual root tensile external stress (vs. extrusion, induced by the vestibular side of the root). The internal root tensile stress was negligible, as opposed to the extrusion movement described above. The MinP correctly described the external compressive stress display in both the tooth and dentine. The internal pulp chamber stress was present on the lingual–mesial and occlusal walls. The internal root canal middle and cervical thirds stress was present with higher extension than the VM and T display, which was clinically and biomechanically incorrect. The depth of higher external stress that was prone to resorptive processes was reduced for both the Min and Max P criteria.

Quantitatively, no significant differences were visible, except for the low amount of tensile stress.

*Rotation* (Figure 4). Qualitatively, the T and VM criteria displayed in the vestibular wall of the pulp chamber showed higher stress in the dentine structure, as well as on the lingual and occlusal ones (Figure 4E). The 4 N orthodontic force applied to the bracket directly induced the color-coded yellow pulpal stress, which was prone to internal pulpal chamber resorption. The coronal external stress seemed to be the highest among the five movements. The internal root canal stress was lower than the coronal one (mostly blue-green, Figure 4E) induced in the lingual cervical third, as it was less prone to resorption. The depth of high red-orange stress manifested in the external dentine structure remained low, following the expected clinical biomechanical behavior. The tooth and dentine stress distribution showed a consistency in patterns (Figure 4A,C,D).

The MaxP tensile stress distribution of high red-orange (Figure 4E) seemed to be the highest of all four criteria, signaling high risks of internal resorption on the vestibular wall of the pulp chamber. Moreover, the internal stress in the root canal was present in both the middle and cervical third and was also prone to internal resorption. Nevertheless, there were some inconsistencies between the stress distribution in the tooth vs. dentine structure, where high stresses are present, signaling potentially less accuracy for this brittle-like criterion.

The MinP compressive stress is lower in the internal dental pulp chamber and root canal than in the other three criteria, indicating no signs of potential resorption.

Quantitatively, the rotation movement displayed the second-highest amounts of stress (T and VM being the highest) after translation, among the five movements, seeming to confirm that rotation was more prone to internal resorptive processes in the pulp chamber.

*Tipping* (Figure 5). Qualitatively, both T and VM displayed a similar external color-coded stress display in the dentine. However, the internal stress in the pulp chamber, despite being present on the lingual–mesial wall, was less extended in the T than in VM, as a sign of the differences in design (T was considered safer than VM). The above-mentioned stress seems to be induced by the external lingual cervical third root stress. The root seems to be free of any stress in both criteria. The MaxP criteria displayed tensile stress on the external vestibular cervical and middle thirds of the root, but with no impact on the interior surfaces of the pulp chamber and root canal, thus having no internal resorptive risks. Nevertheless, the criteria displayed color-coded inconsistencies when assessing the tooth structure, as well as when displaying the extended tensile stress on the external vestibular root surface. Similar inconsistencies were visible for the MinP criteria. The compressive stress seemed to influence both the internal surface of the pulp chamber and the cervical third of the root canal. For both the MinP and MaxP criteria, the pulpal chamber stress seemed to be induced by the external root cervical third distal/vestibular stress, as seen in Table 2.

*Translation* (Figure 6). Qualitatively, T and VM criteria displayed extended color-coded internal pulp chamber stress affecting the occlusal, lingual, and vestibular walls. This internal stress display seems to originate in the lingual–distal external root cervical third stress. The external dentine structure stress is the second largest among the five movements after rotation; nevertheless, the internal pulpal chamber stress is the highest. The internal root canal resorptive process seems to be less intense than the coronal ones. The quantitative amounts of pulp chamber internal stress are almost double that of the external dentinal surface. The same pattern is visible for the brittle-like failure criteria. The tensile MaxP induces internal pulp chamber stress, similar to VM and T, but more extended. The same pattern could be seen for the internal root canal surface. For both stressed areas, the origin is in the external lingual cervical third of the root. Nevertheless, some inconsistencies between the tooth structure and dentine structure stress displays are visible, leading to less accuracy than with the previous above-mentioned criteria. The MinP compressive criteria displayed little internal pulp chamber and root canal stress.

Once correlated (Figure 2, Figure 3, Figure 4, Figure 5 and Figure 6 and Table 2), the ductile failure criteria seem to display the biomechanical behavior of orthodontic movements more accurately in the color-coded internal stress display.

## 4. Discussion

This study continues a previous study investigating the external orthodontic resorptive risks and the failure criteria to be employed to obtain accurate biomechanical behavioral results during the orthodontic movements [48]. The internal resorptive risks were assessed for the intact periodontium by using four failure criteria that were mostly used in the dental studies; the brittle-like tensile MaxP, compressive MinP, ductile-like overall Von Mises and shear Tresca [34,35,36,37,38]. The numerical studies are the only workable possibility in conducting this type of research [2,37]. The FEA provides a countless number of simulations due to the possibility of changing the boundary conditions and the anatomical architecture of the models, simulating various types of movements, as well as various clinical scenarios, which, for the study of such small tissues, are impossible in Vivo conditions [2,13,36,37,41,42]. Moreover, it is the only non-invasive test that allows for internal stress visualization. For this reason, it only needs a single 3D anatomically accurate model. To enhance the accuracy, we chose nine models. Nevertheless, the main limit of the study was related to the impossibility of reproducing the clinical situations identically [1,2,37,40]. Another issue that must be acknowledged is the fact that resorption is a multifactorial biological process, and this stress distribution assessment is only one of them.

It must be acknowledged that numerical studies were conceived for the engineering field, where the situations, materials, and interactions have fewer variables and complexity than in the human body, and to have accurate results, considerations must be closely followed. These are mainly related to the failure criteria, boundary conditions, and anatomical accuracy [36], but are rarely followed in current dental numerical studies, with an obvious impact on their accuracy [34,35,36,37,38].

Due to these issues, there are no other studies investigating the differences between these four failure criteria except our previous reports [34,35,36,37,38]. Moreover, there are only a few studies regarding the internal [20] and external orthodontically induced resorption, most of which are numerical analyses and belong to our team [34,35,48]; thus, there were difficulties in performing direct correlations [4,12,13,14,15,16,17,18,19,44].

The color-coded stress display (Figure 2, Figure 3, Figure 4, Figure 5 and Figure 6) showed that, regardless of the failure criteria used to assess external and internal orthodontically induced stress, the dentine structure must always be separately analyzed to be able to identify the areas that are more prone to suffering resorptive processes. This observation is in line with our previous reports [34,35,48] but contradicts older studies [3,10,45,46,47,50,51,52,53].

The external high stress concentrations (red-orange) are limited to isolated islands that are mainly located in the cervical third of the root (Figure 2C,D, Figure 3C,D, Figure 4C,D, Figure 5C,D and Figure 6C,D), while the internal stress seems to be less intense (yellow-green). Thus, 4 N seems to be less prone to inducing general internal resorptive processes than external, in line with a previous report [48]. Other previous numerical analyses reported equivalent results for 0.6 and 1.2 N [35].

Nevertheless, when closely analyzing each movement, some particular areas could have suffered from localized islands of orthodontic internal resorption, with an impact on the orthodontic treatment planning and possible need for endodontic treatment [21,22,23,24,25,26,27,28,29,30,31,32]. The resorption process is acknowledged to be the result of localized stress concentrations [54] induced by the applied force, anatomical morphology, and movements, and is closely correlated with the individual susceptibility [35]. Moreover, Figure 2E–G, Figure 3E–G, Figure 4E–I, Figure 5E–G and Figure 6E–H showed the internal absorption–dissipation pattern of orthodontically induced force in both external and internal dentinal surfaces, in line with our previous reports regarding the dentine role of dispersing the coronal applied forces [34,35,36,37,38,48].

Thus, the extrusion and intrusion movements induced moderate pulp chamber internal stress in the lingual–mesial and occlusal walls, which were prone to localized resorptive processes, in line with other reports [52,53,55]. However, stress seemed to be the result of the dissipation of the cervical third of the root external stress. No differences were displayed by the VM and T ductile-like criteria when displaying the biomechanical behavior of structures in extrusion and intrusion, in line with [34,35,36,37,38,48]. Nevertheless, inconsistencies were displayed when brittle-like MinP and MaxP criteria were used, with an impact on the behavioral accuracy (since dentine is considered to be ductile-like), in line with our previous report [48]. The extrusion movement seemed to induce both pulp chamber and cervical and middle thirds root canal internal stress, while the intrusion induced mostly pulp chamber stress, seeming less prone to resorptive processes, in line with [35].

An older report [20] investigating an artificial simulated internal resorption (idealized anatomy lower premolar, root canal filled with gutta and MTA, 300 N occlusal force, Von Mises, intact periodontium, 271,837 tetrahedral elements, 414,930 nodes) reported higher vestibular stress when compared with the lingual side for the cervical and middle thirds of the root, with the external surface being red-orange (74.32–107 MPa), and yellow (57.8–83.1 MPa) and the internal surface stresses being red-orange (150.5 MPa) cervical, (381.1 MPa) middle thirds. These results were quantitatively significantly higher than the 0.245–0.682 MPa (VM)/0.285–0.716 MPa (T) MPa herein reported for dentine and our previous reports [34,35,36,37,38,48]. Moreover, these quantitative values [20] exceeded the 29–73.1 MPa reported as maximum shear stress for dentine [60], which seemed to induce resorptive processes, unsupported by clinical practice. On the other side, the qualitative display showed extremely high color-coded stresses in the entire tooth structure [20], while in our study, we found the higher stress only in the dentine structure (in line with previous reports [34,35,36,37,38,48]), with the same root vestibular side spread, but lesser intensity. The above-mentioned differences are issued by the idealized anatomy vs. clinical reality, the simulated internal resorptions, and the reduced number of elements and nodes.

Rotation seems to be prone to internal pulp chamber resorption, directly induced by the applied force on the bracket, exhibiting high amounts of quantitative stress. Nevertheless, it is less prone to internal root canal resorption. Rotation was also reported to be extremely stressful for dental tissues by other numerical studies [45,46,47,50].

Tipping seems to be the movement that is least prone to internal resorption, with no radicular internal stress, but with a certain pulp chamber resorptive risk, in line with a previous study [48]. An older numerical simulation [10] associated with an in vivo study (lower first premolars, tipping, 0.245- and 2.2 N for 12 weeks) reported localized islands of external resorption on the entire root surface, but with no other similarities other than the root surface (lingual and vestibular, as herein).

Translation seems to induce the highest amount of internal pulp chamber stress (Table 2), closely followed by rotation, despite being less extended on the surface. The internal resorptive risks over the pulp chamber induced by those two movements seem to be higher than for the other three. There are differences regarding the pulp chamber stress origin; if the translation originated from the cervical third external root stress, the rotation was directly induced by the applied force. These issues are of importance from a clinical point of view when conceiving the treatment plan [5,6,7,8,9,10,11,33], especially when the dental tissues previously suffered from occlusal trauma and direct/indirect pulp capping [4,5,34,35,36,37,38,39,48].

Another study [10] correlating the in vivo and numerical analysis of external orthodontic resorption reported the role of the anatomical individualities and reactivity in the internal and external resorptive processes and their unpredictability [5,8,9,10,11,33,39,50], suggesting the need for a separate assessment of each tooth, which is also in line with our report.

When analyzing the various patterns provided by ductile and brittle-like criteria, noticeable qualitative inconsistencies arose from differences in how the tissue’s biomechanical behavior was described in mathematical and physical terms. Brittle materials suffered little or no deformation (low absorption–dissipation ability) when subjected to force; stress propagates, leading to yielding and fracture. In contrast, ductile materials experienced recovery deformations long before yielding occurred [34,35,36,37,38]. Dental tissues are more like ductile materials (with a certain brittle flow mode), which, when subjected to very small forces (e.g., from an engineering perspective, a 4 N force behaves similarly to 1 N), induce minimal deformations and displacements [34,35,36,37,38]. Furthermore, the internal tissular micro-architecture [34,42,59,60,61,62] demonstrates the ductility of these tissues [42,57,58,59,60]. The dentine’s absorption–dissipation ability was reported to be between 98.1 and 99.97% of the stress from the applied orthodontic force, with only 0.46–2.38% reaching the pulpal tissue [34,35], reflecting its clinical biomechanical behavior. Therefore, the inconsistencies mentioned above, when MinP and MaxP describe the biomechanical stress behavior, are related to the mathematical design of the failure criteria, resulting in less accurate outcomes despite their use in dental research [61]. Nevertheless, a recent review of numerical dental studies on dentine [61] reported the common use of a combination of brittle (MaxP) and ductile (VM) failure criteria, along with the opinion that dentine is a brittle solid, which contradicts clinical knowledge and overlooks the principles of anatomy and the engineering field of FEA. Our research (herein also) [34,35,36,37,38,48] contradicts this study [61], reporting that the ductile-like criteria are more accurate and proper for dental tissues [38].

There are also visible differences induced by the type of analyzed stress (overall vs. shear) as well as design characteristics that are visible when assessing the difference between VM and T (more intense, extended red-orange islands in VM). The Von Mises is specifically designed for isotropy and to be more accurate (i.e., from the mathematical point of view, yielding depends on the distortion energy) in predicting the biomechanical behavior [34,35,36,37,38,48]. On the other hand, the Tresca is more conservative (i.e., mathematically inside the VM oval interval, and predicting the failure earlier) and is thus safer for human tissues, since the clinical internal and external resorptive mechanisms involve multiple factors besides the amount of applied orthodontic force [34,35,36,37,38,48].

The boundary assumptions related to homogeneity, isotropy, and linear elasticity were assumed by most of the dental numerical studies [4,10,12,13,43,45,46,47,49], since under extremely small forces, with deformations and displacements being barely visible, they were correctly assumed. It must be acknowledged that, from an engineering point of view, 4 N is considered an extremely small force; thus, the above-mentioned boundary assumptions are correct. Moreover, our previous numerical simulations reported similar qualitative results for 0.6, 1.2, and 4 N [34,35,48].

Another issue that must be acknowledged is related to the fact that the internal resorptive processes are also influenced by the pathological status of the dental pulp, especially if associated with orthodontic treatment. Thus, if a previous occlusal trauma history existed, the intrusion/extrusion movements could, in fact, be more prone to resorptive processes [21,22,23,24,25,26,27,28,29,30,31,32]. There are numerical reports of intrusion leading to resorptive risk [52,53,55] in line with this. If a history of coronal pulp dental treatment existed (e.g., direct/indirect pulp capping), the rotation and translation could be more prone to pulp chamber resorption, confirming other clinical reports [21,22,23,24,25,26,27,28,29,30,31,32]. The origins of stress spread in the dentine structure could directly influence the orthodontic plan and justify the additional need for endodontic treatment.

The accuracy of the numerical analysis depends on the anatomical correctness of the 3D models. Thus, an accurate model needs to be based on a CBCT scan (with a small voxel size), with a large number of nodes and elements (e.g., significant accuracy differences between a fixed mesh size of 0.5 mm [4,12,13] and a variable 0.08–0.116 mm herein; automated [4,12,13] vs. manual 3D model segmentation). Nevertheless, despite the above-mentioned, there are numerical reports that still use the idealized anatomical models, which could impact the results [56], with many inconsistencies being reported [50,51,52,53]. We found no reports arguing for the use of a certain failure criterion (except for our previous work [34,35,36,37,38,48]). Most of the numerical studies used only one 3D model (as required, since the method allows for multiple simulations by changing the experimental conditions), mostly reconstructed based on idealized anatomy, with an influence on the experimental results (stress display—as above-mentioned) [34,35,36,37,38,48]. Our study, to enhance the accuracy of the results, used multiple 3D models. Another issue that needs to be acknowledged is the fact that this study assessed intact tooth and dentine structures in the context of intact periodontium. Changes in the tooth structure will induce (depending on their extent) different stress displays, as the numerical simulations regard the external [4,12,13,14,15,16,17,18,19] and internal [20] resorptions; thus, there is a need for more numerical simulations with various levels of bone and tissue loss.

## 5. Conclusions

1. A total of 4 N of orthodontic force could induce orthodontic internal resorption in the pulp chamber (lingual–mesial, vestibular, and occlusal walls), as well as in the middle and cervical thirds of the root canals.
2. Among the five movements, the translation and rotation are more prone to internal pulp chamber resorption (vestibular, occlusal, lingual–mesial walls). However, there is a difference between the two movements regarding the origin of the stress. In the rotation, this was directly induced by the force applied over the bracket, while in translation, the origin of the stress was from the lingual cervical third area.
3. The intrusion and extrusion movements are more prone to root canal cervical and middle thirds’ (vestibular and proximal walls) internal resorptive processes (as a direct result of the external cervical vestibular-localized high stresses induced by the movement).
4. Tipping seems to be the least prone to internal resorption, with a certain pulp chamber lingual–mesial wall risk, because of the internal stress spread originating from the root external cervical third stress.
5. The ductile-like failure criteria seem more accurate when used for the assessment of the internal orthodontically induced resorption than the brittle-like ones.

## 6. Clinical Implications

There are a limited number of reports concerning orthodontically induced internal resorption. Since studying this condition in vivo is clinically impossible, numerical analyses provide the only feasible approach for such studies. To ensure accuracy, a numerical study requires appropriate criteria and a clear rationale for selecting the failure criteria. Despite the significant differences in their accuracy, the four most commonly used failure criteria have not been compared or correlated in the existing research. Our study indicated that ductile-like Tresca and Von Mises criteria appear to be the most precise in characterizing biomechanical behavior during orthodontic movements. These movements demonstrate a specific pattern of stress distribution in both the pulp chamber and root canals, which can predispose to internal resorptive processes. Understanding these patterns, as well as identifying the areas that are under the greatest stress, can improve the planning and outcomes of orthodontic treatment and help to prevent complications such as external and internal resorptions. Thus, knowing which movements are more prone to coronal pulp chamber or root canal radicular internal resorption can make a difference during the treatment. Moreover, the aim of our study is not only to produce data regarding the areas that are prone to resorption, but also to create a method that can be easily used in everyday clinical treatment. Additionally, clinically, teeth and surrounding tissues often experience prior trauma or dental procedures like occlusal trauma or direct/indirect pulp capping, which influence the pulps and tooth’s response to orthodontic forces. Consequently, knowing which movements are more likely to cause radicular or pulpal chamber internal resorption enables the application of preventive measures to achieve optimal results. Overall, this study offers benefits for both clinical practice and future research.

## Figures and Tables

**Table 1:** Physical properties of materials.

Materials	Young’s Modulus, E (GPa)	Poisson Ratio, ʋ	Refs.
Enamel	80	0.33	[34,35,36,37,38,48]
Dentin/Cementum	18.6	0.31	[34,35,36,37,38,48]
Pulp and NVB	0.0021	0.45	[34,35,36,37,38,48]
PDL	0.0667	0.49	[34,35,36,37,38,48]
Cortical bone	14.5	0.323	[34,35,36,37,38,48]
Trabecular bone	1.37	0.3	[34,35,36,37,38,48]
Stainless-steel bracket (Cr-Co)	218	0.33	[34,35,36,37,38,48]

**Table 2:** Quantitative mean amounts of stress (in KPa) produced by 4 N in the dentine structure for the five movements, using the four failure criteria (1 KPa = 0.001 MPa = 1.00 × 10^−3^ MPa).

Movement	Failure Criteria	Component	Apical	Middle	Cervical	Coronal
**extrusion**	**MinP/**Compressive	I	−15.20	−15.20	−70.20	−70.20
		E	−126.10	−126.10	−565.90	**39.91**
	**MaxP/**Tensile	I	307.70	377.90	377.90	167.90
		E	378.00	518.30	728.10	448.00
	**T**/Shear	I	285.30	285.30	500.20	214.50
		E	288.50	288.50	716.60	359.30
	**VM/**Overall	I	244.25	244.25	433.40	185.80
		E	245.23	245.23	682.60	310.40
**intrusion**	**MinP/**Compressive	I	−307.40	−376.50	−376.50	−237.60
		E	−379.20	−519.80	−728.80	−379.80
	**MaxP/**Tensile	I	70.50	70.50	180.20	125.10
		E	348.60	348.60	567.80	127.30
	**T/**Shear	I	285.30	285.30	500.20	214.50
		E	288.50	288.50	716.60	359.30
	**VM/**Overall	I	244.25	244.25	433.40	185.80
		E	245.23	245.23	682.60	310.40
**rotation**	**MinP/**Compressive	I	−40.36	−181.20	−624.10	−624.10
		E	−182.30	−182.30	−1068.10	−735.10
	**MaxP/**Tensile	I	88.15	178.40	540.60	812.50
		E	179.30	270.10	904.00	904.00
	**T/**Shear	I	376.70	376.70	753.10	914.60
		E	472.10	472.10	1131.10	1131.10
	**VM/**Overall	I	326.70	326.70	652.20	816.00
		E	409.10	409.10	980.20	980.20
**tipping**	**MinP/**Compressive	I	−37.50	−37.50	−115.70	−193.40
		E	−428.10	−428.10	−739.10	−428.10
	**MaxP/**Tensile	I	174.70	174.70	174.70	**174.70**
		E	175.70	287.60	287.60	62.80
	**T/**Shear	I	197.10	197.10	197.10	295.30
		E	394.10	394.10	788.10	394.10
	**VM/**Overall	I	171.20	171.20	171.20	256.20
		E	343.10	343.10	685.10	343.10
**translation**	**MinP/**Compressive	I	−20.18	−20.18	−264.10	**−264.10**
		E	−143.10	−143.10	−1242.10	−143.10
	**MaxP/**Tensile	I	107.90	354.40	354.40	**847.50**
		E	108.90	725.30	1341.10	232.10
	**T/**Shear	I	112.4	222.5	778.3	**1000.2**
		E	333.6	445.6	1335.1	445.5
	**VM/**Overall	I	107.20	214.70	750.70	**858.10**
		E	214.60	322.60	1181.00	429.10

I—internal dentine structure and E—external dentine structure; gray and bold marks – brittle criteria inconsistencies.

## Data Availability

The original contributions presented in this study are included in the article. Further inquiries can be directed to the corresponding authors.

## References

[1] Akgün H., Kalyoncuoğlu E. (2025). Mechanical behavior of external root resorption cavities restored with different materials: A 3D-FEA study. BMC Oral Health.

[2] Aktas T., Kosar T. (2025). Finite Element Analysis of Stress Distribution in Various Sizes Internal Root Resorption Cavities Filled with Different Materials. Aust. Endod. J..

[3] Askerbeyli Örs S., Küçükkaya Eren S. (2023). Effects of different treatment modalities on biomechanical behavior of maxillary incisors with external invasive cervical resorption at different progression levels. Dent. Traumatol..

[4] Aslan T., Esim E., Üstün Y. (2025). Stress distribution in restored mandibular molars with external cervical resorption: A finite element analysis. Odontology.

[5] Patel S., Saberi N., Pimental T., Teng P.H. (2022). Present status and future directions: Root resorption. Int. Endod. J..

[6] Kalra S., Gupta P., Tripathi T., Rai P. (2020). External apical root resorption in orthodontic patients: Molecular and genetic basis. J. Fam. Med. Prim. Care.

[7] Bayir F., Bolat Gumus E. (2021). External apical root resorption after orthodontic treatment: Incidence, severity and risk factors. J. Dent. Res. Dent. Clin. Dent. Prospect..

[8] Zhang X., Zhou H., Liao X., Liu Y. (2022). The influence of bracket torque on external apical root resorption in bimaxillary protrusion patients: A retrospective study. BMC Oral Health.

[9] Cheng L.L., Turk T., Elekdag-Turk S., Jones A.S., Petocz P., Darendeliler M.A. (2009). Physical properties of root cementum: Part 13. Repair of root resorption 4 and 8 weeks after the application of continuous light and heavy forces for 4 weeks: A microcomputed-tomography study. Am. J. Orthod. Dentofac. Orthop..

[10] Zhong J., Chen J., Weinkamer R., Darendeliler M.A., Swain M.V., Sue A., Zheng K., Li Q. (2019). In vivo effects of different orthodontic loading on root resorption and correlation with mechanobiological stimulus in periodontal ligament. J. R. Soc. Interface.

[11] Zhang X., Li M.Q., Guo J., Yang H.W., Yu J., Li G.J. (2022). An analysis of the optimal intrusion force of the maxillary central incisor with root horizontal resorption using the finite element method and curve fitting. Comput. Methods Biomech. Biomed. Engin..

[12] Düzgün S., Esim E., Aslan T., Avcı A.T.E. (2025). Finite element analysis of stress in mandibular molars repaired after fractured instrument removal. BMC Oral Health.

[13] Celebi S., Sazak Ovecoglu H. (2024). Evaluating the Restoration of External Root Resorption Under Biomechanical Stress: A Finite Element Analysis. Cureus.

[14] Manaktala M., Taneja S., Bhalla V.K. (2024). Stress distribution in endodontically treated external cervical resorption lesions restored with MTA and biodentine—A finite element analysis. J. Oral Biol. Craniofacial Res..

[15] Çoban Öksüzer M., Şanal Çıkman A. (2024). Evaluation of Fracture Strength after Repair of Cervical External Resorption Cavities with Different Materials. J. Endod..

[16] Sousa J., Azevêdo A.B., Santos R., Silva M., Farias Z., Sobral A.P. (2024). Survival of teeth with external cervical resorption after Internal and External Repair: A Systematic Review. J. Clin. Exp. Dent..

[17] Su R., Li S., Wang W. (2024). Effect of high trimline aligners on distalizing mandibular molars: A three-dimensional finite element study. Eur. J. Med. Res..

[18] Su R., Sun J., Li S., Wang W. (2025). Biomechanical effects of aligner trimline design and intrusion protocol on mandibular anterior teeth: A finite element study. BMC Oral Health.

[19] Yilmaz A., Kabakci A., Helvacioglu Yigit D. (2025). Impact of treatment modalities on stress distribution in maxillary incisors with varying levels of external cervical resorption: A finite element analysis. BMC Oral Health.

[20] Aslan T., Üstün Y., Esim E. (2019). Stress distributions in internal resorption cavities restored with different materials at different root levels: A finite element analysis study. Aust. Endod. J..

[21] Javed F., Al-Kheraif A.A., Romanos E.B., Romanos G.E. (2015). Influence of orthodontic forces on human dental pulp: A systematic review. Arch. Oral Biol..

[22] Bauss O., Rohling J., Meyer K., Kiliaridis S. (2009). Pulp vitality in teeth suffering trauma during orthodontic therapy. Angle Orthod..

[23] Bauss O., Schäfer W., Sadat-Khonsari R., Knösel M. (2010). Influence of orthodontic extrusion on pulpal vitality of traumatized maxillary incisors. J. Endod..

[24] Bauss O., Röhling J., Sadat-Khonsari R., Kiliaridis S. (2008). Influence of orthodontic intrusion on pulpal vitality of previously traumatized maxillary permanent incisors. Am. J. Orthod. Dentofacial Orthop..

[25] Bauss O., Rohling J., Rahman A., Kiliaridis S. (2008). The effect of pulp obliteration on pulpal vitality of orthodontically intruded traumatized teeth. J. Endod..

[26] Strobl H., Haas M., Norer B., Gerhard S., Emshoff R. (2004). Evaluation of pulpal blood flow after tooth splinting of luxated permanent maxillary incisors. Dent. Traumatol..

[27] Emshoff R., Emshoff I., Moschen I., Strobl H. (2004). Diagnostic characteristics of pulpal blood flow levels associated with adverse outcomes of luxated permanent maxillary incisors. Dent. Traumatol..

[28] Chen E., Abbott P.V. (2009). Dental pulp testing: A review. Int. J. Dent..

[29] Farughi A., Rouhani A., Shahmohammadi R., Jafarzadeh H. (2021). Clinical comparison of sensitivity and specificity between sensibility and vitality tests in determining the pulp vitality of mandibular premolars. Aust. Endod. J..

[30] Balevi B. (2019). Cold pulp testing is the simplest and most accurate of all dental pulp sensibility tests. Evid. Based Dent..

[31] Mainkar A., Kim S.G. (2018). Diagnostic Accuracy of 5 Dental Pulp Tests: A Systematic Review and Meta-analysis. J. Endod..

[32] Patro S., Meto A., Mohanty A., Chopra V., Miglani S., Das A., Luke A.M., Hadi D.A., Meto A., Fiorillo L. (2022). Diagnostic Accuracy of Pulp Vitality Tests and Pulp Sensibility Tests for Assessing Pulpal Health in Permanent Teeth: A Systematic Review and Meta-Analysis. Int. J. Environ. Res. Public Health.

[33] Deng Y., Sun Y., Xu T. (2018). Evaluation of root resorption after comprehensive orthodontic treatment using cone beam computed tomography (CBCT): A meta-analysis. BMC Oral Health.

[34] Moga R.A., Olteanu C.D., Botez M.D., Buru S.M. (2023). Assessment of the Orthodontic External Resorption in Periodontal Breakdown-A Finite Elements Analysis (Part I). Healthcare.

[35] Moga R.A., Delean A.G., Buru S.M., Botez M.D., Olteanu C.D. (2023). Orthodontic Internal Resorption Assessment in Periodontal Breakdown-A Finite Elements Analysis (Part II). Healthcare.

[36] Moga R.-A., Olteanu C.D., Delean A.G. (2024). The Amount of Orthodontic Force Reaching the Dental Pulp and Neuro-Vascular Bundle During Orthodontic Movements in the Intact Periodontium. Medicina.

[37] Moga R.-A., Olteanu C.D., Delean A.G. (2024). The Importance of Boundary Conditions and Failure Criterion in Finite Element Analysis Accuracy—A Comparative Assessment of Periodontal Ligament Biomechanical Behavior. Appl. Sci..

[38] Moga R.-A., Olteanu C.D., Delean A.G. (2024). Periodontal Breakdown, Orthodontic Movements and Pulpal Ischemia Correlations—A Comparison Between Five Study Methods. J. Clin. Med..

[39] Chan E., Darendeliler M.A. (2005). Physical properties of root cementum: Part 5. Volumetric analysis of root resorption craters after application of light and heavy orthodontic forces. Am. J. Orthod. Dentofacial Orthop..

[40] Zhao D., Xue K., Meng J., Hu M., Bi F., Tan X. (2023). Orthodontically induced external apical root resorption considerations of root-filled teeth vs vital pulp teeth: A systematic review and meta-analysis. BMC Oral Health.

[41] Maravić T., Comba A., Mazzitelli C., Bartoletti L., Balla I., di Pietro E. (2022). Finite element and in vitro study on biomechanical behavior of endodontically treated premolars restored with direct or indirect composite restorations. Sci. Rep..

[42] Chun K., Choi H., Lee J. (2014). Comparison of mechanical property and role between enamel and dentin in the human teeth. J. Dent. Biomech..

[43] Kabakci A., Yilmaz A., Helvacioglu-Yigit D., Nawar N.N., Kim H.C. (2025). Thermal Behaviour of Teeth with Internal Root Resorption During Obturation and Enhancing Thermal Simulations: A Finite-Element Analysis. Int. Dent. J..

[44] Jain A., Prasantha G.S., Mathew S., Sabrish S. (2021). Analysis of stress in periodontium associated with orthodontic tooth movement: A three dimensional finite element analysis. Comput. Methods Biomech. Biomed. Eng..

[45] Wu J., Liu Y., Li B., Wang D., Dong X., Sun Q., Chen G. (2021). Numerical simulation of optimal range of rotational moment for the mandibular lateral incisor, canine and first premolar based on biomechanical responses of periodontal ligaments: A case study. Clin. Oral Investig..

[46] Wu J., Liu Y., Wang D., Zhang J., Dong X., Jiang X., Xu X. (2019). Investigation of effective intrusion and extrusion force for maxillary canine using finite element analysis. Comput. Methods Biomech. Biomed. Engin..

[47] Wu J.L.L.Y., Peng W., Dong H.Y., Zhang J.X. (2018). A biomechanical case study on the optimal orthodontic force on the maxillary canine tooth based on finite element analysis. J. Zhejiang Univ. Sci. B.

[48] Moga R.-A., Olteanu C.D., Delean A.G. (2026). Orthodontically Induced External Root Resorption: A Finite Element Analysis. J. Clin. Med..

[49] Rodrigues Fonseca Tavares A., Aurelio de Carvalho M., Cardoso Lazari-Carvalho P., Rodrigues de Araújo Estrela L., Santos de Freitas Silva B., Antoninha Del Bel Cury A., Rodrigues Araújo Estrela C. (2025). Teeth with external apical root resorption under orthodontic movement: An in silico analysis on stress and displacement. J. Orofac. Orthop. = Fortschritte Kieferorthopadie.

[50] Wu A.T., Turk T., Colak C., Elekdag-Turk S., Jones A.S., Petocz P., Darendeliler M.A. (2011). Physical properties of root cementum: Part 18. The extent of root resorption after the application of light and heavy controlled rotational orthodontic forces for 4 weeks: A microcomputed tomography study. Am. J. Orthod. Dentofacial Orthop..

[51] Field C., Ichim I., Swain M.V., Chan E., Darendeliler M.A., Li W., Li Q. (2009). Mechanical responses to orthodontic loading: A 3-dimensional finite element multi-tooth model. Am. J. Orthod. Dentofacial Orthop..

[52] Hohmann A., Wolfram U., Geiger M., Boryor A., Kober C., Sander C., Sander F.G. (2009). Correspondences of hydrostatic pressure in periodontal ligament with regions of root resorption: A clinical and a finite element study of the same human teeth. Comput. Methods Programs Biomed..

[53] Hohmann A., Wolfram U., Geiger M., Boryor A., Sander C., Faltin R., Faltin K., Sander F.G. (2007). Periodontal ligament hydrostatic pressure with areas of root resorption after application of a continuous torque moment. Angle Orthod..

[54] Dindaroğlu F., Doğan S. (2016). Root Resorption in Orthodontics. Turk. J. Orthod..

[55] Minch L.E., Sarul M., Nowak R., Kawala B., Antoszewska-Smith J. (2017). Orthodontic intrusion of periodontally-compromised maxillary incisors: 3-dimensional finite element method analysis. Adv. Clin. Exp. Med..

[56] Flatten J., Gedrange T., Bourauel C., Keilig L., Konermann A. (2024). The Role of Bone and Root Resorption on the Biomechanical Behavior of Mandibular Anterior Teeth Subjected to Orthodontic Forces: A Finite Element Approach. Biomedicines.

[57] Jang Y., Hong H.T., Roh B.D., Chun H.J. (2014). Influence of apical root resection on the biomechanical response of a single-rooted tooth: A 3-dimensional finite element analysis. J. Endod..

[58] Giannini M., Soares C.J., de Carvalho R.M. (2004). Ultimate tensile strength of tooth structures. Dent. Mater..

[59] Kailasam V., Rangarajan H., Easwaran H.N., Muthu M.S. (2021). Proximal enamel thickness of the permanent teeth: A systematic review and meta-analysis. Am. J. Orthod. Dentofac. Orthop..

[60] Konishi N., Watanabe L.G., Hilton J.F., Marshall G.W., Marshall S.J., Staninec M. (2002). Dentin shear strength: Effect of distance from the pulp. Dent. Mater..

[61] Ordinola-Zapata R., Lin F., Nagarkar S., Perdigão J. (2022). A critical analysis of research methods and experimental models to study the load capacity and clinical behaviour of the root filled teeth. Int. Endod. J..

[62] Proffit W.R.F.H., Sarver D.M., Ackerman J.L. (2012). Contemporary Orthodontics.

